# The Gut Microbiome Dependency Continuum in Drug Discovery: A Unified Pharmacology Framework Linking Clinical Drugs, Natural Products, and Engineered Microbial Therapeutics

**DOI:** 10.3390/biotech15020043

**Published:** 2026-06-10

**Authors:** Solomon Habtemariam

**Affiliations:** Pharmacognosy Research & Herbal Analysis Services UK, 124 City Road, London EC1V 2NX, UK; s.habtemariam@herbalanalysis.co.uk

**Keywords:** drug discovery, gut microbiome, natural products, faecal microbiota transplantation, programmable microbial systems, engineered probiotics, CRISPR-based microbiome editing, microbiome-responsive drug delivery systems, synthetic microbial consortia, microbiome therapeutics

## Abstract

Highlighting its pivotal role in modern pharmacology, the gut microbiome is emerging as a key determinant of drug efficacy, toxicity, and bioavailability. This review proposes the Gut Microbiome Dependency Continuum, a four-layer framework describing progressively deeper levels of microbiome involvement in drug discovery and therapeutic function. The first layer, intact functional microbiome-dependent therapeutics and includes interventions such as faecal microbiota transplantation and defined microbial consortia. The second layer, microbiome-modulated approved drugs include widely used therapeutics whose pharmacokinetics or pharmacodynamics are strongly influenced by microbial metabolism. Examples include metformin, irinotecan, levodopa, and digoxin, where gut microbial interactions influence efficacy, toxicity, and inter-individual variability in treatment outcomes. The third layer, microbiota-transformable natural products, encompasses dietary and plant-derived compounds such as polyphenols, ginsenosides, alkaloids, fibres, isoflavones, lignans, and glucosinolates. Their biological activity depends on microbial biotransformation into bioactive metabolites. The fourth layer, engineered microbiome therapeutics, includes synthetic biology approaches such as programmable microbial systems, engineered probiotics, CRISPR-based microbiome editing, and microbiome-responsive drug delivery systems. It also includes synthetic microbial consortia, enabling targeted sensing, therapeutic delivery, and ecological reprogramming of gut microbial communities. Altogether, these layers define a continuum in which the gut microbiome evolves from a passive modulator to an essential metabolic organ and ultimately a programmable therapeutic platform. The article provides an integrated framework for microbiome-informed drug discovery. It also supports the development of precision, ecology-aware, and engineered microbial therapeutics.

## 1. Introduction

The human gut microbiome is a metabolically active ecosystem whose genetic repertoire exceeds that of the host by several orders of magnitude. In the intestine alone, studies from as early as the 1970s showed that the human microbiota contains roughly ten times more bacterial cells than human host cells [1]. Recent metagenomic studies have now shown the presence of some 3.3 million unique genes in the human gut microbiome, which translates to 150 times more genes than our own genome [2]. Beyond projection and estimates, the Human Microbiome Project revealed a unified sequence catalogue representing over 200,000 genomes and 171 million protein sequences of the human gut microbiome [3,4]. Unlike the human genome, however, the composition of the gut microbiome is not inherited as a composite of parental genes but is acquired over time from birth. It is shaped predominantly by environmental exposures (e.g., diet, lifestyle, and interspecies competition) while also undergoing constant interaction with host genetics [5,6,7]. As a result, the extent of variation observed in the human microbiome between individuals vastly surpasses the relatively minor genetic differences found within the human genome. The composition and diversity of the gut microbiota vary further depending on health status, with distinct microbial patterns observed in conditions such as cancer, diabetes, and IBD, among others. Hence, the complexity of the gut microbiome, along with its immense diversity and variability, poses a significant challenge to implementing a universal, one-size-fits-all approach to microbiome-based therapeutics in human disease.

The gut microbial community is coming to be recognised as an active biochemical system rather than a passive environmental factor. It acts as a dynamic metabolic interface that transforms xenobiotics, produces beneficial metabolites and bioactive enzymes, and regulates host physiology to influence health, diseases, and therapeutic outcomes. Based on principles from pharmacogenomics, early pharmacological models assumed that drug response was governed mainly by host enzymes, transporters, and genetic variation. This framework was supported by evidence showing that genetic polymorphisms in drug-metabolising enzymes (e.g., CYP450 enzymes, dihydropyrimidine dehydrogenase, uridine diphosphate glucuronosyltransferase, glutathione *S*-transferase, sulfotransferase, etc), transporters like ATP-binding cassette sub-family B member 1, and drug targets can markedly influence drug efficacy, metabolism, and toxicity [8,9]. However, this host-centred assumption is widely challenged by accumulating evidence demonstrating that microbial metabolism functions as an independent and, in some cases, dominant determinant of drug outcomes.

In response to this paradigm shift, the emerging field of pharmacomicrobiomics has been established to systematically characterise drug–microbiome interactions. Recent multi-omics and experimental studies demonstrated that the gut microbes can activate, inactivate, toxify or detoxify a wide range of pharmaceutical agents through enzymatic biotransformation and metabolic competition [10,11,12]. As detailed in the following sections, microbial enzymatic activity can fundamentally alter clinical efficacy and toxicity profiles of drugs such as metformin, irinotecan, and levodopa. At the same time, dietary compounds and natural products undergo extensive microbial biotransformation, yielding metabolites with distinct pharmacological activity when compared to their parent structures. More recently, synthetic biology approaches have enabled the engineering of microbial systems capable of producing therapeutic compounds which respond to disease signals, or modulating host physiology in situ. Despite this expanding knowledge base, the field lacks a unified conceptual structure that integrates clinical pharmacology, natural product chemistry, and engineered microbial systems into a single continuum. Here, the microbiome dependency continuum is proposed as a unifying framework that organises microbiome involvement in drug discovery along a graded spectrum of increasing biological relevance and engineering control. It spans from minimal microbial influence on drug action to complete microbiome-mediated dependency in which microbial metabolism, ecology, and host–microbe interactions are essential determinants of therapeutic efficacy and safety.

## 2. Overview of the Gut Microbiome Dependency Continuum

**Figure 1:**
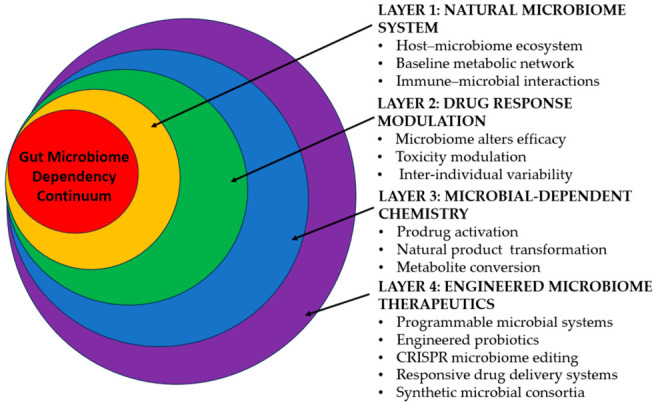
The Gut Microbiome Dependency Continuum in drug discovery. Concentric overlapping circles illustrate increasing microbiome involvement in therapeutic design and outcome. Layer 1 represents the intact host–microbial ecosystem regulating baseline metabolic and immune interactions. Layer 2 shows microbiome-modulated drug responses, where microbial variation alters drug efficacy and toxicity. Layer 3 depicts microbiome-dependent chemistry, in which microbial metabolism transforms xenobiotics and natural products into active or inactive metabolites. Layer 4 represents engineered microbiome therapeutics, including programmable microbes, engineered probiotics, CRISPR-based editing, microbiome-responsive delivery systems, and synthetic microbial consortia. Altogether, the model highlights a continuum from natural microbiome function to fully engineered therapeutic platforms.

The central premise of the Gut Microbiome Dependency Continuum is that drug action can be understood as a graded function of microbiome involvement rather than a binary interaction. This review reflects the growing evidence that microbial contributions to pharmacology are neither uniform nor optional, but instead vary systematic across therapeutic modalities, chemical classes, and host contexts. At one extreme are therapies that depend entirely on intact microbial ecosystems for efficacy, while at the other end are engineered systems in which microbial behaviour is deliberately programmed to achieve defined therapeutic outcomes (Figure 1). Between these extremes lie the widely used clinical drugs whose efficacy is modulated by microbial metabolism, as well as natural products whose bioactivity emerges only after microbial transformation within the gut environment (Figure 1). Furthermore, this framework captures the fact that microbiome influence is not static but dynamically shaped by ecological structure, metabolic capacity, and host–microbe interactions, which collectively determine whether microbial activity is incidental, modulatory, or essential to drug function. In the following sections, the structured four conceptual layers of this continuum are presented with levels of microbiome integration into pharmacological function ranging from passive microbial exposure effects to fully engineered microbiome-dependent therapeutic systems.

## 3. LAYER 1—Highest Level of Microbiome Dependency: Requirement for an Intact Functional Ecosystem

At the highest level of microbiome dependency, therapeutic efficacy is contingent upon the presence of an intact, diverse, and functionally competent microbial ecosystem, such that disruption of this ecosystem results in complete or near-complete loss of therapeutic effect. In this regime, the gut microbiome is not merely a modulator of pharmacological response but an essential biological substrate required for clinical efficacy. This represents a shift from viewing microbes as auxiliary contributors to recognising them as indispensable components of therapeutic function, particularly in interventions where ecological restoration or microbial community function is the direct mechanism of action.

The most well-established clinical example of microbiome-based therapy is faecal microbiota transplantation (FMT) in recurrent *Clostridioides difficile* infection (rCDI), where restoration of a disrupted gut microbiota can achieve high cure rates under clinical conditions [13]. *C. difficile* is an anaerobic, Gram-positive, spore-forming bacterium that produces potent exotoxins, but its ability to colonise and cause disease is closely linked to disruption of the intestinal microbiota. Infection typically follows exposure to broad-spectrum antibiotics, which deplete commensal bacterial populations and impair colonisation resistance [14]. Under physiological conditions, the gut microbiota restricts pathogen expansion through nutrient competition, production of antimicrobial metabolites, and modulation of host immune responses [15]. When antibiotic-induced dysbiosis disrupts these protective mechanisms, it creates a permissive environment that enables *C. difficile* spore germination and subsequent vegetative outgrowth.

A key mechanistic link between microbiome disruption and CDI is the alteration of bile acid metabolism. Commensal gut bacteria normally convert primary bile acids into secondary bile acids, which inhibit the growth of *C. difficile*. However, earlier studies [14,15] clearly established that antibiotic-induced dysbiosis impairs this conversion, leading to accumulation of primary bile acids that promote spore germination and vegetative outgrowth. Studies in the 1990s [16] showed that the shift in the intestinal metabolome creates a permissive environment for colonisation and enhances toxin production including principal virulence factors, toxin A and toxin B. These toxins inactivate the Rho family GTPases in intestinal epithelial cells, resulting in cytoskeletal disorganisation, disruption of tight junctions, and epithelial barrier breakdown [17]. Both toxins also simultaneously trigger immune cells to release proinflammatory cytokines (e.g., interleukin (IL)-1β, IL-6, IL-8, IL-17A, and IL-16) which are now believed to serve as biomarkers for CDI and predictors of disease severity [18]. Studies have further shown that rCDI infection arises from the persistence of spores together with an inability to re-establish a healthy gut microbiome following antibiotic therapy [19]. Although standard treatments such as vancomycin or fidaxomicin effectively suppress vegetative *C. difficile*, they can further disrupt commensal microbial communities and fail to eliminate spores, thereby predisposing patients to recurrence [14]. This limitation has driven the development of microbiome-based therapeutics that aim to restore ecological balance and colonisation resistance rather than directly targeting the pathogen.

Historically, FMT involved the administration of fresh or frozen donor stool via colonoscopy, enema, or nasogastric tube. Over the past decade, however, this approach has evolved into regulated, standardised live biotherapeutic products designed to improve safety, reproducibility, and scalability. The transition from a clinical procedure to drug-like biologics was realised between 2022 and 2023 with the emergence of the first FDA (the US Food and Drug Administration)-approved microbiome therapeutics. Two notable examples are Rebyota (RBX2660), approved as a rectal suspension, and Vowst (SER-109), approved as oral capsules. Clinical evidence supporting the approval of Rebyota (RBX2660), a microbiota-based live biotherapeutic derived from screened donor stool, demonstrated that restoration of a diverse microbial community can significantly reduce rCDI. Administered as a single-dose rectal suspension, Rebyota was shown in randomised controlled trials (RCTs) to improve clinical outcomes when compared with placebo, with sustained responses observed in a substantial proportion of treated patients. It received FDA approval in 2022 as the first licenced faecal microbiota-based therapy for the prevention of rCDI in adults following antibiotic treatment [20]. Clinical studies further indicate a treatment success rate of approximately 70% at 8 weeks, with durable remission maintained in over 90% of responders over longer follow-up periods, highlighting its effectiveness as a standardised alternative to traditional FMT [21]. Similarly, clinical evidence supporting the approval of oral SER-109, which consists of faecal-derived purified *Firmicutes* bacterial spores, demonstrated that it is well-tolerated and provides significant therapeutic benefit in patients with rCDI [22,23]. Marketed as Vowst, SER-109 received FDA approval in 2023, for the prevention of rCDI in adults. Above all, clinical outcome assessments of capsule-based FMT have shown cure rates of approximately 80–90% in rCDI, highlighting the effectiveness of orally delivered microbiome therapeutics [24].

Of the pioneering work that led to the proof of concept of such therapeutics is a study by van Nood et al. [13] with a small group of 16 patients which showed that FMT was significantly more effective than vancomycin, and increased faecal bacterial diversity, similar to that in healthy donors. They also noted an increase in *Bacteroidetes* species and clostridium clusters IV and XIVa and a decrease in *Proteobacteria* species. These outcomes were supported by multiple clinical trials, but what is worth mentioning as a critical assessment of delivery method was an RCT by Kao et al. [25]. The study established that FMT via oral capsules was not inferior to delivery by colonoscopy for preventing rCDI infection over a 12-week study period. Moreover, the cure or no occurrence rate at 12 weeks was 89.5% for the capsule group and 96.6% for the colonoscopy group. Another pioneering study assessment was based on frozen versus fresh FMT comparison in an RCT study by Lee et al. [26] and Youngster et al. [27] in adults with recurrent or refractory CDI. They reported that the use of frozen FMT did not result in a worse proportion of clinical resolution of diarrhoea suggesting frozen FMT, preparations and storage of this therapeuticsare equally acceptable. As described above, studies since then have established that FMT using freeze-dried capsules has a similar safety and effectiveness profile when compared with colon-FMT, without the procedural risks of colonoscopy [24]. Hence, the past decade has seen the transition from traditional donor stool FMT to regulated live biotherapeutic products, as evidence by the FDA-approved drugs Rebyota and Vowst. These therapies aim to deliver defined or standardised microbial communities with improved safety, reproducibility, and scalability. Readers can review the extensive successful clinical trials on these therapies but some studies with no significant reduction in rCDI are also reported, underscoring the complexity of microbiome therapeutics. For example, an RCT by Drekonja et al. [28] showed the therapy did not reduce CDI recurrence or death at 56 days. This suggest that therapeutic efficacy depends on factors such as microbial composition, viability, dosing, and host–microbiome interactions, rather than the concept of FMT alone.

The VE303 represents a further evolution of FMT into a fully defined, rationally designed microbiome therapeutic. Unlike traditional FMT, which transfers a complex and variable community of donor microbes, VE303 microbiome therapy is a synthetic bacterial consortium composed of eight selected strains of commensal *Clostridia* commensal class, chosen for their ability to restore key ecological and metabolic functions of the healthy gut microbiome. It was developed specifically to treat rCDI by mimicking the protective effects of FMT in a controlled and reproducible way. It is known to act through competitive exclusion as well as bile acid metabolism restoration. Being an engineered microbial ecology, however, it does suffer from donor variability. A phase 2 RCT study with 79 participants showed its benefit on the prevention of CDI [29]. In a phase 2 clinical trial, VE303 has been shown to reduce the odds of recurrent rCDI by >80% when compared with the placebo group [30]. The study further allowed prediction of CDI recurrence based on a higher level of primary bile acid levels, and lower levels of secondary bile acid and short-chain fatty acids (SCFAs). Hence, taurochenodeoxycholic acid was shown to predict recurrence, while lithocholic acid, deoxycholic acid, hexanoate and isovalerate predicted non-recurrence. These findings are in line with the various studies where microbial-derived secondary metabolites inhibit different stages of the *C. difficile* life cycle from spore germination through vegetative growth and toxin production (see review articles [31,32,33]). In healthy volunteers, VE303 dosing has been shown to allow optimal colonisation when applied over multiple days after vancomycin pretreatment and it promotes the establishment of a microbiota even under cases of colonisation resistance [34]. Hence, the successful outcomes were associated with engraftment of the administered strains, supporting the idea that targeted ecological restoration rather than broad microbial transfer is sufficient to confer protection. Conceptually, VE303 sits between traditional FMT and single-strain probiotics: it is a defined, multi-strain live biotherapeutic product designed based on ecological principles. The approach addresses key limitations of FMT, including donor variability, safety concerns, and lack of standardisation, while preserving the multi-organism functionality needed for durable microbiome restoration. As such, VE303 exemplifies the shift toward precision microbiome therapeutics, where specific microbial consortia are engineered to treat disease in a predictable and scalable manner.

The FMT has also emerged as a potential microbiome-based therapeutic for IBD, particularly ulcerative colitis, where dysbiosis is thought to contribute to aberrant mucosal immune activation. Multiple RCTs over the past decade [35,36,37] have demonstrated that FMT can induce clinical and endoscopic remission in subsets of patients. Mechanistic and clinical analysis of the STOP-Colitis trial based on FMT further demonstrate that the approach achieved clinical response rates as high as 75% depending on delivery route and regimen [38]. These findings are supported by meta-analyses of double-blind RCTs, demonstrating that FMT can induce clinical and endoscopic remission in active ulcerative colitis, with a safety profile comparable to placebo [37]. However, more recent meta-analyses reported variable efficacy across studies and patient subgroups, underscoring the heterogeneity and evolving nature of FMT-based therapy in IBD (e.g., [39]). The compelling evidence now demonstrates that FMT is clinically effective in inducing remission in IBD, but variability in outcomes highlights the importance of donor selection, microbial composition, and treatment protocols, further underscoring the evolving and not universally established setting of FMT-based therapy. In this context, the microbiome functions as an essential organ-like system, and therapeutic success depends not on targeting individual microbial components but on reconstructing the ecological and metabolic networks that underpin host–microbe homeostasis. Hence, despite the progress toward defined multi-strain consortia, substantial variability in engraftment and clinical response, particularly in complex conditions such as IBD, remains a key unresolved challenge.

While FMT is most clearly validated in CDI, it has also catalysed the development of alternative microbiome-based and cell-derived therapeutics that aim to deliver defined organisms or functions rather than whole-community transfer. One such approach is the use of probiotic strains with targeted metabolic activity, exemplified by *Clostridium butyricum* MIYAIRI 588. This is a butyrate-producing anaerobe that has been used clinically in parts of Asia. It has been shown that MIYAIRI 588 can modify the gut microbiome under antibiotic-induced dysbiosis and increased the butyric acid and oleic acid content while also inducing anti-inflammatory effects [40]. Another notable proof-of-concept study was that by Depommier et al. [41], which investigated the effect of *Akkermansia muciniphila* supplementation in overweight and obese individuals. Remarkably, the pasteurised (non-viable) form of the bacterium improved insulin sensitivity, reduced insulinaemia, and lowered markers of inflammation when compared with placebo. This suggests that specific bacterial components rather than live colonisation alone can mediate therapeutic effects. In experimental animals, oral *C. butyricum* administration ameliorates intestinal inflammation and enhances barrier function by modulating the gut microbiota and its metabolites, while also alleviating diarrhoea symptoms [42]. Mechanistically, restoration of SCFAs levels along with other metabolic pathways, including bile acid and fatty acid metabolism, have been noted. This therapeutic approach represents a shift toward postbiotic therapy, where bacterial-derived molecules are harnessed for clinical benefit. Butyrate is a key SCFA that supports epithelial barrier integrity and exerts anti-inflammatory effects and its benefit via supplementation with *C. butyricum* in antibiotic-associated diarrhoea and other pathological conditions have been extensively researched in recent years.

In metabolic disorders such as type-2 diabetes (T2D), early randomised studies [43,44,45] showed that transfer of microbiota from lean donors can transiently improve insulin sensitivity, supporting a causal role for the microbiome in metabolic regulation. In oncology, preclinical and translational studies indicate that FMT from responders can enhance the efficacy of immune checkpoint inhibitors in cancers such as melanoma (e.g., [46,47]), suggesting that microbial composition modulates antitumour immunity. The phase 2 FMT-LUMINate trial further showed that FMT from immunotherapy responders can safely modulate the gut microbiome and may enhance responsiveness to immune checkpoint inhibitors in patients with advanced non-small cell lung cancer and melanoma [48]. However, the clinical benefit was variable, reinforcing the fact that microbiome modulation is a promising but still experimental strategy requiring further optimisation. Another exploratory trail was that of the phase 1 PERFORM trial which demonstrated that FMT combined with immunotherapy is feasible and safe in patients with metastatic renal cell carcinoma. The preliminary evidence from the study suggest that microbiome modulation may enhance responses to immune checkpoint inhibitors [48,49]. Similarly, emerging research widely supports a role for the gut–brain axis in neurodegenerative disorders such as Parkinson’s disease (PD) and Alzheimer’s disease (AD), where alterations in the intestinal microbiome are thought to influence neuroinflammation, protein aggregation, and disease progression. Preclinical studies have provided strong mechanistic evidence, as shown by the study of Sampson et al. [50], which demonstrated that germ-free or antibiotic-treated mice overexpressing α-synuclein exhibit reduced motor deficits and neuroinflammation. On the other hand, colonisation with microbiota from patients with PD exacerbated the pathology, establishing a causal link between gut microbes and disease phenotype. In AD models, microbiome manipulation has similarly been shown to modulate amyloid-β (Aβ) deposition and microglial activation (e.g., [51]). Specifically, these data showed that a germ-free amyloid precursor protein (APP) transgenic mice show a marked reduction in cerebral Aβ pathology when compared with conventionally raised mice that possess a normal intestinal microbiota. However, colonisation of germ-free mice with microbiota from APP transgenic donors markedly increases Aβ deposition, whereas microbiota from wild-type donors induces only a modest rise in cerebral Aβ levels. The study by Jiang et al. [52] showed that FMT improved cognitive performance and reduced Aβ pathology in a mouse model of AD, likely through modulation of gut microbiota and attenuation of neuroinflammation. Translational and early clinical studies extend these findings in recent years. A small-scale trials and case series have reported that FMT can alter gut microbial composition and may improve gastrointestinal and, in some cases, neurological symptoms in PD (e.g., [53,54]). These studies, however, are very limited by small sample sizes and lack of controls. Although clinical evidence in AD remains sparse, pilot studies suggest that microbiome-targeted interventions can influence systemic inflammation and cognitive-associated biomarkers. It can thus be summarised that scientific evidence so far supports a unifying therapeutic strategy whereby restoration or engineering of a healthy microbiome may correct disease-associated dysbiosis and downstream metabolic or immune dysfunction. Unlike the well-established efficacy in CDI, however, FMT in these complex, multifactorial diseases remains inconsistent and largely experimental. Clinical responses remain variable, reflecting key limitations such as donor heterogeneity, unstable microbial engraftment, lack of standardisation, and strong host dependence (e.g., diet, genetics, baseline microbiota). These challenges drive the shift from crude FMT toward defined, mechanism-based microbial consortia and precision microbiome therapeutics. The overall mechanistic base of intact functional microbiome drug discovery approach is summarised in Table 1.

## 4. Layer 2—Microbiome-Modulated Approved Drugs

A far larger proportion of clinically approved drugs fall into the category of microbiome-modulated therapeutics, in which microbial metabolism significantly alters drug pharmacokinetics or pharmacodynamics without being the primary intended mechanism of action. A prominent example is metformin, whose antidiabetic efficacy extends beyond direct activation of host AMP-activated protein kinase to include substantial microbiome-mediated mechanisms. An early mechanistic study demonstrated that metformin treatment significantly remodels the gut microbial ecosystem, increasing the relative abundance of mucin-degrading and metabolically beneficial taxa such as *A. muciniphila* and SCFA-producing bacteria [55]. These compositional shifts are associated with increased production of microbial metabolites, including SCFAs, which have been shown to improve insulin sensitivity and energy homeostasis through activation of G-protein-coupled receptors such as GPR41 and GPR43 [56,57]. The study by Godet et al. [58] similarly showed that metformin improves glucose tolerance in diabetic mice partly by reshaping the gut microbiota and enhancing gut functions such as mucus production and glucagon-like peptide-1 (GLP-1)-related signalling. These microbiota- and gut-driven changes correlate with the metabolic benefits, supporting a key role for the antidiabetic action of metformin via the gut–microbiome axis. Furthermore, metformin alters intestinal bile acid pools and signalling, enhancing GLP-1 secretion via farnesoid X receptor (FXR) and TGR5 (Takeda G-protein-coupled receptor 5)-dependent pathways [59]. In an RCT study in treatment-naive T2D patients using metformin, a significant change in the gut microbiome composition and increased abundance of certain bacteria linked to improved glucose metabolism were noted [60]. Furthermore, transferring microbiota from metformin-treated individuals into germ-free mice improved glucose tolerance, suggesting that at least part of antidiabetic effect of metformin was mediated indirectly through gut microbiome changes. This also positions metformin as a canonical example of a microbiome-modulated therapeutic rather than a purely host-targeted drug.

**Figure 2:**
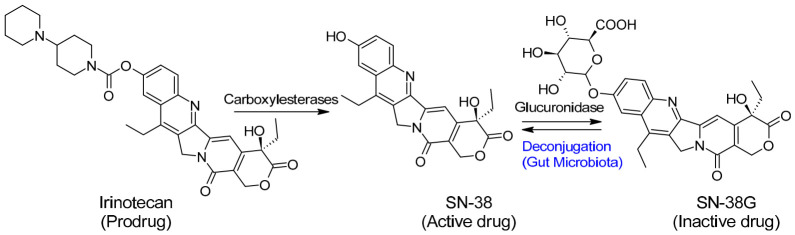
The metabolic fate of irinotecan in the liver and intestine. Irinotecan is a prodrug which requires conversion to its active form (SN-38) by hepatic carboxylesterases. Its detoxification also occurs in the liver through glucuronidation into SN-38G through the action of uridine diphosphate glucuronosyltransferases. While excreted via bile, the gut microbiota through the action of β-glucuronidases convert SN-38G back into active/toxic SN-38 form leading to diarrhoea and local intestinal damage.

In the case of irinotecan, gut microbial enzymes play a direct and clinically significant role in its toxicity through enterohepatic reactivation. Irinotecan is converted in the liver to its active metabolite SN-38 (7-ethyl-10-hydroxycamptothecin), which is subsequently detoxified by hepatic glucuronidation to SN-38G (SN-38 glucuronide) before biliary excretion. However, microbial β-glucuronidases within the intestinal lumen can deconjugate SN-38G back into the active SN-38 form (Figure 2), resulting in local accumulation of the cytotoxic compound and damage to the intestinal epithelium [61]. Yamamoto et al. [62] used an anaerobic mixed culture of rat caecal microorganisms to show that intestinal microflora can metabolise SN-38 through bacterial β-glucuronidase activity, and confirming that gut bacteria influence both the drug’s intestinal activation and its associated gastrointestinal toxicity. This microbial reactivation process is strongly implicated in the development of irinotecan-associated diarrhoea, which is a dose-limiting toxicity that can be severe and treatment-limiting in patients. Bhatt et al. [63] showed that selectively inhibiting gut bacterial β-glucuronidase prevents reactivation of irinotecan metabolites in the intestine, thereby reducing gastrointestinal toxicity and improving the tolerability and overall efficacy of its anticancer chemotherapy. A strong set of original studies supporting this conclusion shows that gut bacterial β-glucuronidase activity directly drives irinotecan-induced intestinal toxicity by reactivating SN-38 in the gut lumen, and that pharmacological inhibition of this microbial enzyme protects the intestinal epithelium without altering systemic antitumour drug exposure or efficacy [64,65]. A follow-up mechanistic experiment in these studies using selective small-molecule inhibitors demonstrates that blocking bacterial β-glucuronidases reduces chemotherapy-associated mucosal damage and weight loss in preclinical models while preserving tumour regression. This reinforces the idea that microbial metabolism can be selectively targeted to mitigate drug toxicity without compromising anticancer activity [61,66]. On these bases, one can conclude that the pharmacology of irinotecan is critically shaped by host–microbiome metabolic interactions within the gut.

**Figure 3:**
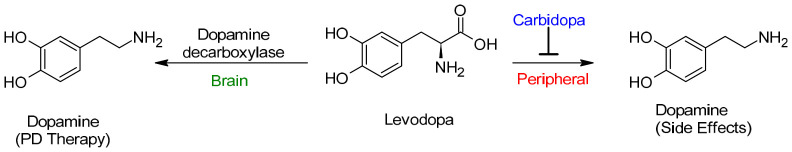
Gut microbial metabolism competes with brain delivery of levodopa. In Parkinson’s disease (PD), orally administered levodopa may reach the brain and be converted to dopamine, while peripheral host and microbial metabolism reduce its availability. Although carbidopa inhibits host aromatic L-amino acid decarboxylase, gut microbes such as *Enterococcus faecalis* and *Eggerthella lenta* metabolise levodopa and its products, limiting systemic uptake.

The pharmacokinetics of levodopa are strongly influenced by gut microbial metabolism, which can reduce systemic drug availability and contribute to inter-individual variability in therapeutic response in PD. Goldin et al. [67] demonstrated in rats that intestinal bacteria significantly contribute to the metabolism of levodopa by converting it into dopamine within the gut lumen before absorption, thereby reducing the amount of levodopa available for systemic uptake and brain delivery. The study also showed that this microbial decarboxylation can be suppressed with antibiotics, leading to increased circulating levodopa levels and enhanced pharmacological availability. Maini Rekdal et al. [68] further showed that gut bacteria reduce levodopa bioavailability through a two-step interspecies pathway in which *Enterococcus faecalis* converts levodopa to dopamine and *Eggerthella lenta* further metabolises it, limiting host drug availability. They also identified a small-molecule inhibitor that selectively blocks the bacterial enzyme responsible for the first step, increasing levodopa levels without affecting host enzymes or systemic drug metabolism [68]. This microbial metabolism not only reduces levodopa bioavailability but also contributes to variability in motor symptom control across patients. In modern anti-parkinsonian therapy, levodopa is commonly co-administered with peripheral enzyme inhibitors such as carbidopa or benserazide to block its peripheral decarboxylation (Figure 3), thereby reducing systemic degradation, increasing central nervous system availability, and improving therapeutic efficacy. Hence, a microbial enzymatic activity can act as a parallel metabolic route that competes with host drug handling, thereby shaping both efficacy and dosing requirements in PD therapy.

Haiser et al. [69] showed that the gut bacterium *E. lenta* can inactivate the cardiac drug digoxin in a strain-dependent manner via a specific reductase enzyme system, leading to a reduction in bioavailability. They also demonstrated that this microbial drug inactivation can be modulated by dietary arginine, which suppresses bacterial expression of the responsible genes and thereby preserves digoxin activity. On the other hand, sulfasalazine, a drug used in the treatment of ulcerative colitis, is cleaved by gut bacterial azoreductases into its active metabolite 5-aminosalicylic acid, making microbial metabolism essential for its therapeutic activation [70]. The study by Zhao et al. [71] further demonstrated that enhancing gut microbial azoreductase activity through a probiotic–drug co-delivery system increases sulfasalazine activation and improves its therapeutic efficacy in IBD. Statins, including simvastatin and rosuvastatin, show variable lipid-lowering efficacy that correlates with gut microbiome composition and bile acid metabolism [72]. Liu et al. [73] further showed that the in vivo rosuvastatin lipid-lowering efficacy was associated with baseline gut microbiome composition, with specific microbial profiles linked to stronger or weaker low-density lipoprotein reduction. The study also suggested that microbial community structure can influence host lipid metabolism and statin response variability. Cyclophosphamide exhibits microbiome-dependent immunomodulatory effects, where gut bacteria enhance antitumour immune responses by promoting Th1/Th17 polarisation [74]. He et al. [75] showed that xylooligosaccharides modulate the gut microbiota and its metabolites, which in turn enhances immune function and counteracts cyclophosphamide-induced immunosuppression. These data reinforced the microbiome’s role in shaping the immunomodulatory effect of xylooligosaccharides. Similarly, the toxicity of 5-fluorouracil is modulated by gut microbial composition and mucosal interactions that influence intestinal inflammation and drug-related metabolic stress [76,77,78]. Antibiotics themselves can also be considered microbiome-interactive drugs: clindamycin and broad-spectrum β-lactams profoundly reshape microbial communities, indirectly influencing susceptibility to secondary infections such as CDI [14]. Even drugs not primarily targeting the gut, such as acetaminophen and sulphonamides, exhibit microbiome-dependent variability in pharmacokinetics, as alterations in gut microbial composition can modify drug biodisposition, hepatic metabolism, and enterohepatic cycling through changes in microbial-derived metabolites and host–microbe co-metabolism [79,80].

Readers should bear in mind that, beyond antibiotics, drugs also act as key ecological modulators of the gut microbiome, representing a reverse pharmacomicrobiomics framework in which xenobiotics actively reshape microbial community structure and function. The commonly used drug classes such as proton pump inhibitors, antipsychotics, and metabolic agents (e.g., metformin) have been shown to alter gut microbial diversity and composition through direct antimicrobial activity and indirect host-mediated effects [81,82]. Longitudinal evidence further suggests that these perturbations can persist beyond treatment periods, indicating durable microbiome restructuring far beyond transient dysbiosis [83]. Functionally, such drug-induced shifts influence microbial metabolic pathways, including SCFA production and amino acid fermentation, leading to pharmacological and metabolic outcomes [84].

Overall, Layer 2 demonstrates that the microbiome functions as an unrecognised pharmacokinetic layer embedded within standard drug action. A summary of microbiome-modulated therapeutics outlining their mechanistic and clinical consequences is presented in Table 2.

## 5. Layer 3—Microbiota-Transferable Natural Products

Natural products represent a chemically diverse class of compounds whose pharmacological activity frequently depends on microbial transformation. Unlike many synthetic drugs, these compounds often exhibit low intrinsic bioavailability and require enzymatic modification by the gut microbiota to generate active or more readily absorbed metabolites. In this context, the intestinal microbiome functions as an auxiliary metabolic organ that expands host biochemical capacity through a wide range of reductive, hydrolytic, and fermentative reactions.

### 5.1. Polyphenols

**Figure 4:**
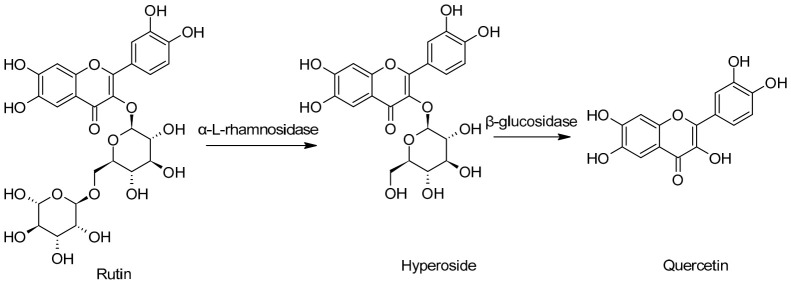
Microbial activation of quercetin glycosides in the gut. Dietary rutin is hydrolysed by gut microbial α-L-rhamnosidase and β-glucosidase to release quercetin. The aglycone is more readily absorbed, leading to increased bioavailability and bioactivity. Other flavonoid glycosides, including quercetin-3-*O*-glucoside and quercetin-3-*O*-rhamnoside, are similarly converted to quercetin by bacterial glycosidases.

Dietary polyphenols exert many of their biological effects through a dynamic and reciprocal interaction with the gut microbiota, rather than acting solely as intact compounds absorbed in the small intestine. A substantial proportion of ingested polyphenols, particularly complex flavonoids and tannins, reach the colon unmetabolised, where they undergo extensive microbial biotransformation. In vitro studies using human faecal microbiota have demonstrated that flavonoids such as quercetin derivatives are degraded into smaller phenolic acids through microbial enzymatic processes including deglycosylation and ring fission [85]. These transformations are critical because the resulting metabolites are often more bioavailable and biologically active than their parent compounds, establishing that microbial metabolism is a key determinant of polyphenol function [86]. For example, polyphenols such as rutin require gut microbial metabolism to become bioactive, as intestinal bacteria cleave its glycosidic bonds to produce metabolites like quercetin, which have greater systemic bioavailability and biological activity (Figure 4). This has been demonstrated in the study by Hanske et al. [87], which showed that human gut microbiota metabolise rutin into quercetin glycosides with distinct bioactivity. The study by Aura et al. [85] further confirmed that colonic bacteria are essential for the conversion of rutin into absorbable phenolic metabolites.

**Figure 5:**
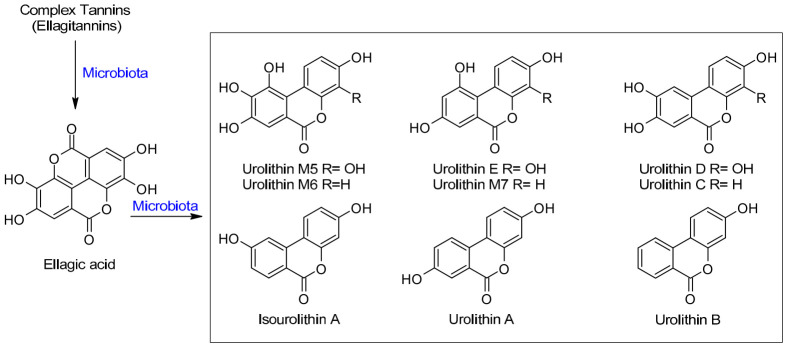
Gut microbial metabolism of ellagitannins to urolithins with progressive dihydroxylation. Dietary ellagitannins are hydrolysed to ellagic acid and further metabolised by gut microbiota into intermediate and final urolithins through sequential dehydroxylation and lactone modification. Progressive loss of hydroxyl groups generates metabolites ranging from highly oxygenated intermediates to the predominant human end products, urolithin A and urolithin B.

A well-characterised example of natural products processing in the gut is the microbial conversion of ellagitannins and ellagic acid into urolithins. In a controlled fermentation study, García-Villalba et al. [88] demonstrated the stepwise production of urolithins by human gut microbiota, involving sequential reactions such as lactone-ring cleavage and dehydroxylation. These metabolites (Figure 4) are not present in the original diet but are generated exclusively through microbial activity. In a further support of this mechanism, Selma et al. [89] identified specific bacterial species, including *Gordonibacter urolithinfaciens*, that are capable of catalysing key steps in urolithin formation. Beltrán et al. [90] further described additional taxa such as *Ellagibacter isourolithinifaciens* that contribute to this metabolic pathway. The dependence on specific microbial taxa leads to inter-individual variability in metabolite production, suggesting that the same dietary intake can result in different physiological outcomes depending on microbiota composition. Beyond this, the study by Tomás-Barberán et al. [91] shows that human gut microbiota consistently converts ellagic acid into urolithins in three distinct metabolic phenotypes (A, B, and 0), reflecting stable inter-individual differences in microbial activity. These phenotypes occur independently of diet, age, or health status, suggesting they are intrinsic microbiota-related traits that may influence responses to ellagitannin-rich foods. Phenotype A predominantly produces urolithin A and phenotype B produces a mixture of urolithins (including urolithins A, B, and isourolithin A), while phenotype 0 shows little or no capacity to produce urolithins at all. The structural variation in urolithins produced by the gut microbiota with variability mostly arising from the degree and pattern of oxygenation in the two aromatic rings is shown in Figure 5.

**Figure 6:**
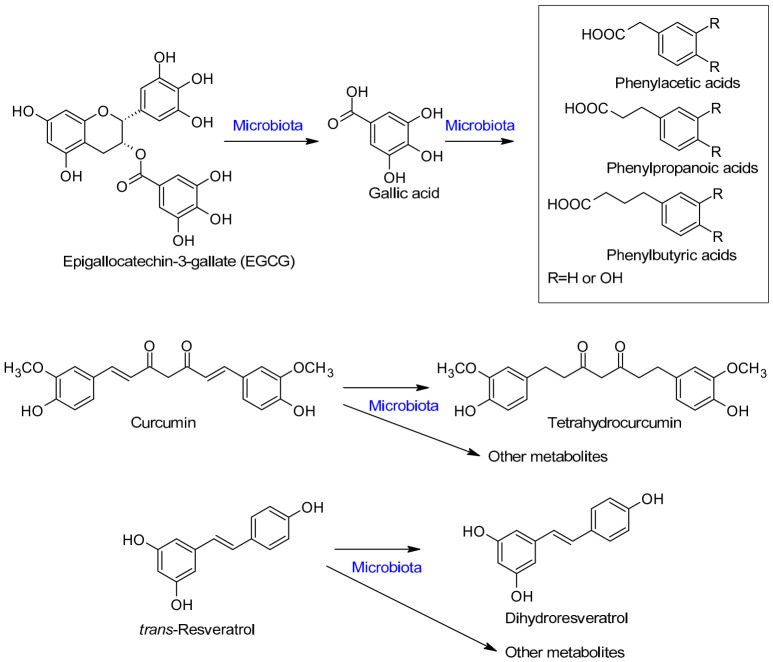
Gut microbiota-mediated biotransformation of major dietary polyphenols into bioactive metabolites. Gut microbiota metabolise curcumin, resveratrol, and epigallocatechin gallate (EGCG) into bioactive metabolites through reduction, hydrogenation, and ring fission reactions. The resulting products include tetrahydrocurcumin, dihydroresveratrol, and phenolic acids, with metabolite profiles varying between individuals according to gut microbial composition and metabolic capacity.

Beyond flavonoids and tannins, other polyphenols such as curcumin, resveratrol, and epigallocatechin gallate (EGCG) also depend on gut microbiota for their biotransformation and bioactivity (Figure 6). For example, Luo et al. [92] and Lou et al. [93] demonstrated that curcumin is extensively metabolised by human intestinal microbiota into tetrahydrocurcumin and several smaller phenolic compounds with altered biological activity, while Bode et al. [94] showed that resveratrol undergoes microbial conversion into dihydroresveratrol, a key bioactive metabolite. Similarly, Takagaki and Nanjo [95] and Liu et al. [96] reported that EGCG is degraded by intestinal bacteria into valerolactones and phenolic acids via ring fission reactions. The studies highlight that the therapeutic effects of diverse polyphenols are strongly influenced by gut microbiota-mediated metabolism, which governs their bioavailability and functional activity.

In addition to being metabolised by gut microbes, polyphenols actively influence the composition and function of the microbiota itself. In vitro fermentation experiments have shown that compounds such as rutin and quercetin can alter microbial growth and metabolic outputs, including the production of SCFAs [97]. Human intervention studies further support this bidirectional relationship; for example, cocoa-derived flavanols have been shown to increase beneficial bacterial populations such as *Bifidobacterium* and *Lactobacillus* while reducing potentially pathogenic species [98]. Similarly, animal and mechanistic studies demonstrate that polyphenol-rich diets can induce significant shifts in microbial community structure, reinforcing their role as modulators of gut ecology [99]. These findings demonstrate that the biological activity of dietary polyphenols emerges from a bidirectional interaction with the gut microbiota. Microbial metabolism transforms polyphenols into bioactive compounds that mediate host effects, while polyphenols simultaneously shape microbial composition and metabolic activity. This interplay establishes the gut microbiota as a central determinant of polyphenol efficacy and supports the concept that these compounds function as microbiota-dependent modulators of host physiology.

### 5.2. Ginsenosides

**Figure 7:**
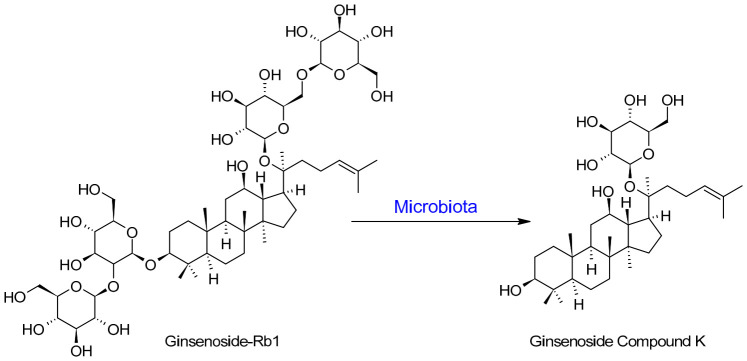
Gut microbiota-mediated biotransformation of ginsenoside Rb1 into the bioactive metabolite ginsenoside compound K. Gut microbial β-glucosidases sequentially remove sugar moieties from ginsenoside Rb1 to produce the more lipophilic and bioactive metabolite compound K. This bioconversion enhances bioavailability and is essential for the in vivo pharmacological activity of ginsenoside Rb1.

A comparable dependence on microbial activation is observed for glycosylated natural products, where sugar moieties limit absorption until they are removed by intestinal microbes. Ginsenosides, the major bioactive constituents of *Panax* species, are triterpenoid saponins typically present as highly glycosylated compounds with low membrane permeability and poor oral bioavailability. Akao et al. [100] demonstrated that intestinal bacteria are required to convert ginsenoside Rb1 into ginsenoside compound K (Figure 7) via sequential deglycosylation, and that compound K appears in plasma only after microbial metabolism. This transformation is mediated by microbial β-glucosidases that sequentially hydrolyse sugar moieties, generating intermediates such as Rd and F2 before producing compound K as the final metabolite. These findings demonstrate that the pharmacological activity of ginsenosides depends largely on microbial biotransformation rather than direct absorption of the parent compounds. Subsequent studies have identified specific bacterial taxa responsible for this biotransformation, providing further mechanistic support for microbiota-dependent activation. For example, Bacteroides JY-6 and *Eubacterium* spp. A-44 were shown to convert ginsenoside Rb1 into compound K through defined enzymatic pathways involving sequential sugar cleavage [101,102]. These studies demonstrated that the presence and abundance of specific gut microbes directly determine the efficiency of compound K production, leading to inter-individual variability in pharmacokinetics and therapeutic response. In vivo experiments further confirmed that compound K is the predominant form detected in systemic circulation following oral administration of ginsenosides, reinforcing the conclusion that microbial metabolism is a prerequisite for bioavailability.

Above all, compound K exhibits significantly greater biological activity than its parent ginsenosides. Experimental studies have shown that it possesses enhanced anti-inflammatory and anticancer properties, including inhibition of NF-κB (nuclear factor kappa-light-chain-enhancer of activated B cells) signalling and induction of apoptosis in tumour cells [103,104]. This mechanism reflects a broader principle applicable to many dietary and plant-derived glycosides. Glycosylation increases molecular size and polarity, reducing passive diffusion across the intestinal epithelium and thereby limiting direct host uptake. Microbial enzymes, particularly glycosidases, overcome this barrier by cleaving sugar moieties and releasing aglycones or simplified derivatives that can be absorbed and further metabolised by host tissues. Consequently, the biological effects of glycosylated natural products emerge from a cooperative interaction between host and microbiota, in which microbial metabolism governs both the extent of absorption and the nature of the active compounds. This paradigm closely parallels the microbiota-dependent activation observed for polyphenols and reinforces the concept that gut microbes play a central role in determining the pharmacological outcomes of dietary phytochemicals.

The translation of ginsenoside Rb1 to bioactive metabolite compound K for consistent human clinical outcomes remains a challenge due to marked inter-individual variability in gut microbiome composition and enzymatic capacity, particularly in taxa expressing β-glucosidase activity [105]. This variability leads to substantial differences in the efficiency and kinetics of the conversion across individuals. Moreover, dietary patterns, especially fibre intake and habitual diet composition, influence microbial community structure and substrate competition, thereby modulating biotransformation potential [106]. These, together with host-related factors such as age, disease state, intestinal transit time, and baseline metabolic status can further contribute to variability in compound K exposure and downstream pharmacodynamic effects.

### 5.3. Alkaloids

**Figure 8:**
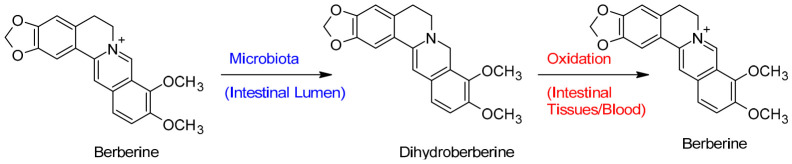
Gut microbiota-mediated conversion of berberine to dihydroberberine and its impact on intestinal absorption. Gut microbial reductases convert berberine to the more lipophilic dihydroberberine, enhancing intestinal absorption. After uptake, host tissues rapidly re-oxidise dihydroberberine back to berberine, improving overall bioavailability and pharmacological efficacy.

Alkaloids also undergo microbiome-dependent transformations that significantly influence their pharmacokinetic properties and systemic activity. A well-characterised example is the isoquinoline alkaloid berberine, which exhibits extremely low intrinsic oral bioavailability despite pronounced metabolic effects in vivo. In a pivotal study, Feng et al. [107] demonstrated that intestinal microbiota convert berberine into dihydroberberine (Figure 8) via nitroreductase-mediated reduction. This metabolite is more lipophilic and exhibits enhanced membrane permeability, allowing it to be absorbed more efficiently across the intestinal epithelium. Once inside the gut, berberine is extensively transformed by intestinal microbiota into dihydroberberine through microbial reductive enzymes, a conversion shown to enhance intestinal absorption and systemic availability. Wei et al. [108] demonstrated that this biotransformation is microbiota-dependent and driven in part by bacterial nitroreductase activity, with antibiotic-mediated depletion of gut bacteria significantly reducing dihydroberberine formation and lowering berberine exposure in vivo. Consistently, Feng et al. [109] further showed that restoration or presence of a functional microbiota increases both metabolite production and pharmacokinetic efficiency, confirming that microbial metabolism is essential for berberine bioavailability and therapeutic action.

The enzymatic basis of this transformation further supports its microbiota dependence. Nitroreductase activity capable of catalysing the reduction in berberine has been identified in multiple gut-associated bacterial taxa, including species within the genera *Bacteroides* and *Enterococcus*, indicating that this is a community-level metabolic function rather than a host-driven process [107]. Complementary pharmacokinetic studies have shown that berberine undergoes extensive first-pass metabolism and exhibits poor intestinal absorption in germ-free or microbiota-disrupted models, reinforcing the requirement for microbial conversion to achieve physiologically relevant systemic levels [110]. These findings collectively demonstrate that the gut microbiota act as a critical determinant of berberine disposition by transiently modifying its physicochemical properties to facilitate uptake.

In contrast to polyphenols and glycosylated natural products, where microbial metabolism often generates entirely new bioactive metabolites, the case of berberine illustrates a distinct mechanism in which microbiota enhance absorption without fundamentally altering the pharmacophore. Nevertheless, microbial metabolism can also modulate downstream metabolic pathways and tissue distribution. For example, studies have shown that berberine alters gut microbial composition and metabolic output, including SCFA production, which may contribute indirectly to its metabolic effects [111]. This suggests a dual interaction in which the microbiota both enable berberine absorption and participate in mediating its systemic activity.

Beyond berberine, microbiota-dependent transformations have been observed for other alkaloids, further supporting the generality of this phenomenon. For instance, the metabolism of plant-derived alkaloids frequently involves microbial reduction, demethylation, and dehydroxylation reactions that alter their bioavailability and activity. Research on indole alkaloids has demonstrated that gut bacteria can convert complex structures into simpler, more absorbable metabolites, thereby influencing their pharmacokinetic profiles [112]. Similarly, microbial biotransformation of dietary alkaloids can generate metabolites with altered receptor-binding properties or enhanced systemic exposure, depending on the structure of the parent compound. Taken together, these findings establish that alkaloids, like polyphenols and glycosylated natural products, are subject to significant microbiota-dependent modulation.

### 5.4. Dietary Fibres

Dietary fibres provide one of the clearest and most extensively studied examples of microbiota-dependent activation of natural products, in which otherwise indigestible substrates are converted into bioactive metabolites that directly regulate host physiology. Non-digestible polysaccharides such as resistant starches, inulin, and other complex plant fibres resist digestion by host enzymes in the upper gastrointestinal tract and instead reach the colon largely intact. They are metabolised by anaerobic gut bacteria through saccharolytic fermentation pathways, yielding SCFAs, primarily acetate, propionate, and butyrate. Experimental studies integrating in vivo and in vitro models has demonstrated that these SCFAs are the principal mediators of fibre-induced health effects, linking microbial metabolism to host lipid metabolism, immune regulation, and energy homeostasis [113]. Mechanistically, SCFA production is a community-level metabolic process involving multiple bacterial taxa and cross-feeding interactions. Primary degraders such as *Ruminococcus bromii* initiate the breakdown of complex polysaccharides into oligosaccharides, which are subsequently utilised by secondary fermenters including *Faecalibacterium prausnitzii* and *Eubacterium rectale* to generate butyrate and other SCFAs [114]. This cooperative metabolism highlights that fibre fermentation is not attributable to a single organism but rather emerges from the collective enzymatic capacity of the gut microbiome. The resulting SCFA profile depends on both substrate composition and microbial community structure, leading to inter-individual variability in metabolic outputs and physiological responses.

The bioactivity of SCFAs arises from both receptor-mediated signalling and epigenetic mechanisms. Butyrate and propionate have been shown to activate G-protein-coupled receptors such as GPR41 and GPR43 on intestinal epithelial and immune cells, thereby modulating inflammatory responses and energy balance [115]. Butyrate also functions as a histone deacetylase inhibitor, altering chromatin structure and gene expression in colonocytes and immune cells, which contributes to enhanced epithelial barrier integrity and anti-inflammatory effects [116]. These dual modes of action provide a direct mechanistic link between microbial metabolism of dietary fibre and host gene regulation.

In vivo studies further support the causal role of microbiota-derived SCFAs in mediating the physiological effects of dietary fibre. Germ-free or antibiotic-treated animal models exhibit markedly reduced SCFA production and fail to display the metabolic and immunological benefits associated with fibre consumption [117]. This demonstrates that the observed effects are microbiota-dependent. Moreover, supplementation with fermentable fibres has been shown to increase circulating SCFAs levels and improve metabolic parameters, including insulin sensitivity and lipid metabolism, in both animal models and human studies [103,117]. These findings confirm that the host lacks the intrinsic capacity to derive significant metabolic benefit from dietary fibre in the absence of microbial fermentation.

Human intervention studies indicate that fermentable fibres such as resistant starch and inulin-type fructans increase SCFA concentrations, but the magnitude and profile of SCFA production response (acetate, propionate, butyrate) depend strongly on fibre type and dose, with resistant starch favouring butyrate production and inulin-type fibres more strongly influencing acetate and propionate pathways [118,119]. Inter-individual variability is once more a dominant feature of this relationship, as baseline microbiota composition, functional capacity, and metabolic network structure significantly modulate fermentation efficiency and SCFA output [120,121]. Recent multi-cohort analyses further confirm that, while fibre supplementation consistently shifts SCFA-associated microbial pathways, the magnitude of metabolite response varies widely between individuals, supporting a microbiome-conditioned rather than universal dose–response model [120,122].

Overall, dietary fibres exemplify a fundamental paradigm in natural product biology: the generation of bioactivity through microbial metabolism. Unlike polyphenols or alkaloids, where microbial transformation modifies existing bioactive scaffolds, fibres are largely inert until converted into SCFAs, which function as key signalling molecules influencing host physiology.

### 5.5. Isoflavones

**Figure 9:**
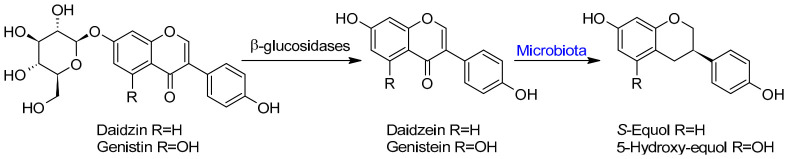
Sequential host and gut microbiota-mediated biotransformation of dietary isoflavones into bioactive metabolites. Intestinal β-glucosidases convert daidzin and genistin into the aglycones daidzein and genistein, which are further metabolised by gut microbiota into bioactive metabolites such as equol and 5-hydroxy-equol. Production of these metabolites varies between individuals according to gut microbial composition and metabolic capacity.

Isoflavones provide a well-defined example of microbiota-dependent metabolic activation, in which gut bacteria convert relatively weakly active dietary compounds into more potent bioactive metabolites. The soy isoflavone daidzein, typically present as a glycoside in food (Figure 9), is first hydrolysed to its aglycone form and then further reduced by specific intestinal bacteria to produce equol. This transformation involves a series of reductive steps, including the formation of intermediates such as dihydrodaidzein and tetrahydrodaidzein, catalysed by bacterial enzymes expressed by a limited subset of gut microbes. Early human studies demonstrated that only approximately 30–50% of individuals harbour the microbial consortia required for equol production, a phenomenon that has been consistently observed across populations and is strongly associated with differences in gut microbiota composition [123,124].

The biological significance of this microbial conversion lies in the markedly enhanced activity of equol when compared to its precursor daidzein. Experimental studies have demonstrated that specific gut bacteria are capable of converting daidzein to equol via defined enzymatic pathways, with isolates such as equol-producing intestinal strains confirming this biotransformation at the microbial level [125]. Further mechanistic study has identified the microbial communities and metabolic pathways responsible for this conversion, showing that equol production depends on the presence of distinct bacterial taxa within the gut ecosystem [126]. In human studies, the ability to produce equol is closely linked to gut microbiota composition, with distinct microbial community profiles determining whether individuals can metabolise dietary daidzein into equol [127]. This variability provides direct evidence that the health effects of isoflavones are not solely determined by dietary intake but depend critically on the metabolic capacity of the gut microbiota.

At the mechanistic level, specific bacterial taxa responsible for equol production have been isolated and characterised, further supporting a causal role for the microbiome. For example, *Slackia isoflavoniconvertens* and *Adlercreutzia equolifaciens* have been shown to carry the enzymatic machinery required to convert daidzein into equol through defined reductive pathways [128,129]. The presence, abundance, and activity of these bacteria determine whether equol is produced in vivo, explaining the observed metabotype differences among individuals. In vivo evidence further confirms the microbiota dependence of this pathway. Antibiotic treatment or germ-free conditions abolish equol production, while colonisation with equol-producing strains restores this metabolic capability, demonstrating a direct causal link between microbial composition and metabolite formation [124]. Altogether, these findings establish that isoflavones function as microbiota-dependent precursors whose biological activity is amplified through bacterial metabolism. This paradigm closely parallels other natural product classes, reinforcing the broader concept that the gut microbiome governs both the qualitative and quantitative efficacy aspect of dietary bioactive compounds.

### 5.6. Plant Lignans

**Figure 10:**
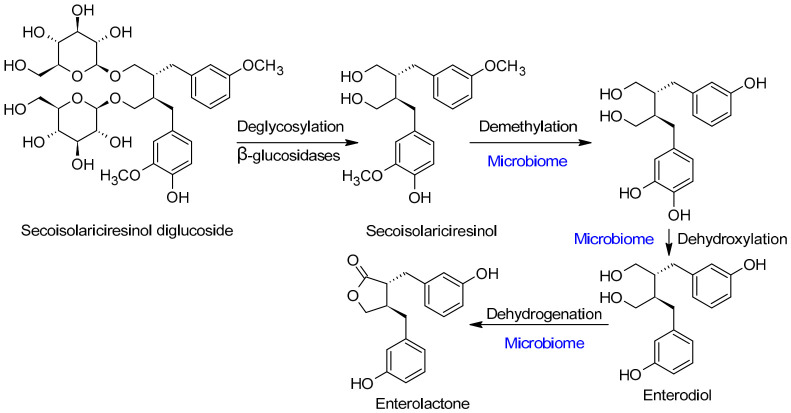
Gut microbiota-mediated conversion of dietary lignans to mammalian lignans enterodiol and enterolactone. Gut microbiota convert secoisolariciresinol diglucoside into the bioactive mammalian lignans enterodiol and enterolactone through deglycosylation, reduction, demethylation, and dehydroxylation reactions. Production efficiency varies between individuals according to gut microbial composition and metabolic capacity.

Plant lignans provide another clear example of microbiota-dependent metabolic activation, in which relatively inert dietary precursors are converted into bioactive compounds with endocrine activity. Lignans such as secoisolariciresinol diglucoside, commonly found in flaxseed and other plant foods, are ingested as glycosylated forms that are poorly absorbed in the small intestine. Upon reaching the colon, they undergo sequential biotransformation by gut bacteria, beginning with deglycosylation to release the aglycone secoisolariciresinol, followed by a series of reductive and dehydroxylation reactions that yield the mammalian lignans enterodiol and enterolactone (Figure 10). Early mechanistic studies demonstrated that these conversions involve specific enzymatic steps, including *O*-demethylation and dehydroxylation, carried out by anaerobic intestinal bacteria [130]. At the microbial level, this transformation is mediated by a consortium of bacterial species rather than a single organism, highlighting the importance of community metabolism. For example, *E. lenta* has been shown to catalyse initial demethylation steps, while *Peptostreptococcus productus participates* in downstream reductions leading to enterodiol formation [130]. Additional species, including *Lactonifactor longoviformis*, are responsible for the final conversion of enterodiol into enterolactone via lactonisation reactions [131]. This stepwise pathway illustrates a coordinated, multi-species metabolic network in which intermediate products are transferred between bacterial taxa through cross-feeding interactions.

Inter-individual variability in enterolignan production further underscores the central role of the gut microbiome. Human metagenomic and metabolomic studies have demonstrated that the capacity to convert dietary lignans into enterodiol and enterolactone varies markedly between individuals and is strongly associated with distinct gut microbial community structures, leading to the classification of enterolignan producer and non-producer phenotypes [132]. This variability is driven by differences in the abundance of lignan-metabolising bacterial taxa, which directly determine the efficiency of microbial biotransformation pathways [133]. Above all, clinical and epidemiological evidence shows that antibiotic exposure significantly reduces circulating enterolactone levels, confirming that disruption of gut microbial activity leads to diminished enterolignan formation in vivo [134]. Taken together, lignans exemplify a broader paradigm in which the gut microbiome converts plant-derived compounds into hormonally active metabolites through coordinated, multi-step biochemical transformations.

### 5.7. Glucosinolates

**Figure 11:**
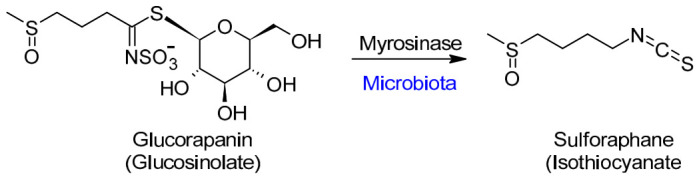
Enzymatic conversion of glucosinolates to isothiocyanates: role of plant and gut microbial myrosinase in sulforaphane formation. Glucoraphanin is hydrolysed to the bioactive isothiocyanate sulforaphane by plant myrosinase or, when plant enzyme activity is reduced, by gut microbial myrosinase-like enzymes. Conversion efficiency varies between individuals according to gut microbial composition and enzymatic activity.

Glucosinolates from cruciferous vegetables are sulphur- and nitrogen-containing glycosides that require enzymatic hydrolysis to generate biologically active isothiocyanates. While plant myrosinase normally catalyses this reaction in intact tissues, food processing and cooking inactivate this enzyme, shifting hydrolysis to the gut microbiota. In vitro and ex vivo human faecal fermentation studies have demonstrated that intestinal bacteria can convert glucoraphanin and related glucosinolates into isothiocyanates such as sulforaphane (Figure 11), confirming microbial involvement in glucosinolate activation [135,136]. Furthermore, mechanistic microbiological studies have identified gut bacterial enzymatic activity capable of driving partial glucosinolate hydrolysis, although conversion efficiency varies significantly between individuals depending on microbiota composition [136]. These microbial-derived isothiocyanates exhibit greater biological activity than their parent glucosinolates, including induction of phase 2 detoxification enzymes and modulation of xenobiotic metabolism pathways, highlighting the functional importance of gut microbiota in their bioactivation.

Earlier studies have also confirmed and extended these findings by identifying variability in microbial conversion efficiency and the structural dependence of glucosinolate metabolism. For example, Shapiro et al. [137] demonstrated in human feeding studies that urinary excretion of isothiocyanate metabolites varies widely between individuals consuming identical glucosinolate-rich foods, indicating that bioactivation depends on inter-individual differences in gut microbial composition and enzymatic capacity. This variability is further supported by in vitro fermentation experiments showing that colonic bacteria can differentially hydrolyse aliphatic, indole, and aromatic glucosinolates, producing distinct isothiocyanate and nitrile profiles depending on substrate structure and microbial community composition [138].

Mechanistically, glucosinolate hydrolysis in the gut is a community-level process involving multiple bacterial taxa with β-thioglucosidase-like activity rather than a single specialist organism. Ex vivo fermentation and microbiome studies have shown that members of the gut microbiota, including *Bacteroides* spp. and *Enterococcus* spp., can contribute to thioglucosidic bond cleavage under anaerobic conditions, generating unstable aglycone intermediates that spontaneously rearrange into isothiocyanates [135,136]. Furthermore, evidence from *Brassica* glucosinolate chemistry demonstrates that the relative formation of isothiocyanates, nitriles, and epithionitriles is strongly influenced by environmental and biochemical conditions such as pH, genotype, and enzymatic context, highlighting the inherent chemical selectivity of glucosinolate breakdown pathways [139]. These findings indicate that glucosinolate metabolism is both microbially mediated and chemically constrained, leading to variable production of bioactive and less bioactive breakdown products.

In vivo human studies further reinforce the physiological relevance of microbiota-dependent glucosinolate metabolism. Controlled feeding trials using broccoli sprouts have demonstrated substantial inter-individual variation in sulforaphane and related isothiocyanate metabolite excretion, with differences strongly associated with gut microbiome composition and specific bacterial taxa [140]. Microbiome profiling in these studies shows that individuals with distinct microbial community structures exhibit significantly different capacities to convert glucoraphanin into bioactive isothiocyanates versus inert nitrile products, directly linking microbial ecology to host exposure [141]. These findings are supported by broader human intervention evidence demonstrating that variability in gut microbial function is a key determinant of systemic isothiocyanate bioavailability following cruciferous vegetable consumption, highlighting the microbiome as a critical modulator of chemoprotective efficacy. The overall role of microbiota in the pharmacological activity of natural products is summarised in Table 3.

## 6. Layer 4—Engineered Microbiome Therapeutics

The landscape of engineered microbiome therapeutics is organised into five interconnected subdomains spanning increasing levels of biological design and control. These include programmable microbial systems, engineered probiotics as living therapeutics, CRISPR (clustered regularly interspaced short palindromic repeats)-based microbiome editing, microbiome-responsive drug delivery systems, and synthetic microbial consortia. The following sections discuss these approaches to reflect a transition from passive microbiome modulation to fully programmable and ecologically engineered systems capable of sensing, computing, delivering therapeutic payloads, and reshaping microbial community structure.

### 6.1. Programmable Microbial Systems and Synthetic Gene Circuits

**Figure 12:**
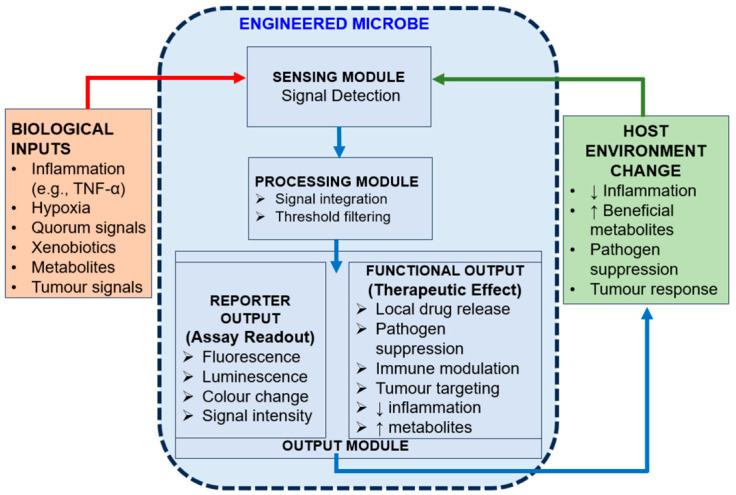
Programmable engineered microbes as synthetic gene circuit platforms for biosensing and therapeutic modulation. Host-derived signals, including inflammatory factors, metabolites, pH, and xenobiotics, are detected by microbial sensing modules and processed through genetic circuits to generate reporter or therapeutic outputs. Feedback from the host environment enables closed-loop regulation, allowing engineered microbes to function as living biosensors and therapeutic agents. Up (↑) or down (↓) arrows denote a significant increase or reduction in the indicated biological activity, respectively.

At the most advanced end of the microbiome–drug interaction continuum, microbial systems can be rationally engineered to function as programmable platforms for drug discovery and evaluation. Synthetic biology enables the construction of genetic circuits that convert biological inputs into measurable outputs, allowing engineered bacteria to act as living biosensors of host physiology and drug action. Early foundational work by Anderson et al. [142] demonstrated that engineered bacteria can selectively localise to tumour environments and be externally regulated to control therapeutic gene expression. More sophisticated gene circuits have been developed since then in which engineered microbes sense endogenous tumour-associated conditions such as hypoxia and quorum signals and trigger coordinated population-level responses, including synchronised lysis for controlled therapeutic release [143,144]. These systems have further been extended beyond therapeutic delivery into functional biosensing and drug discovery applications, where engineered bacterial biosensors act as in vivo reporters of physiological and pharmacological states. For example, engineered *E. coli* Nissle 1917 has been programmed with genetic memory circuits that stably record exposure to inflammation-associated signals in the gut, enabling in vivo retrospective reconstruction of disease activity and environmental exposure [145]. More recent synthetic biology work has expanded this paradigm by using engineered bacterial systems as in vivo metabolic and pharmacological reporters, capable of recording host-associated biochemical signals and reporting on xenobiotic exposure and enzymatic activity in gut-relevant environments, thereby extending functional drug assessment beyond conventional in vitro assays [145,146,147]. Altogether, these studies establish programmable microbial systems as emerging drug discovery tools that integrate biosensing, molecular recording, and functional readouts within living biological environments. A schematic presentation of drug discovery-applicable programmable microbial systems and synthetic gene circuits is presented in Figure 12.

### 6.2. Engineered Probiotics as Living Therapeutics

Engineered probiotic strains represent a key translational application of synthetic biology in which commensal or food-grade microbes are repurposed as in situ drug production and delivery systems. A foundational demonstration of this concept was the engineering of *E. coli* Nissle 1917 to metabolise phenylalanine, leading to significant reduction in systemic phenylalanine levels in preclinical models of phenylketonuria [148]. This established the feasibility of using gut-resident bacteria as continuous metabolic therapies. The study also provided a blueprint for leveraging microbial metabolism to correct host biochemical imbalances in a sustained and localised manner. Subsequent studies have expanded engineered probiotics beyond substrate depletion toward localised biosynthesis and delivery of therapeutic molecules within the gut environment. An early proof-of-concept study demonstrated that *Lactococcus lactis* engineered to secrete interleukin (IL)-10 could reduce intestinal inflammation in murine colitis models while avoiding systemic immunosuppression [149]. Tumas et al. [150] further demonstrated that *E. coli* Nissle 1917 can be repurposed as a localised cytokine delivery platform, producing bioactive IL-2 directly within the tumour microenvironment to enhance antitumour immune responses. This study highlights the potential of engineered microbes to overcome systemic toxicity associated with conventional cytokine therapies by enabling site-specific immunomodulation. It also shows the expanding scope of microbiome-based strategies in drug discovery and cancer immunotherapy.

Over the past five years, however, the field of engineered probiotics has undergone a significant transition from proof-of-concept systems to clinically oriented live biotherapeutic platforms. Engineered *E. coli* Nissle strains have been advanced as delivery vehicles for immunomodulatory molecules and enzymes across multiple disease areas, including cancer and metabolic disorders. For example, a first-in-human study of an engineered *E. coli* Nissle strain (SYNB1891) demonstrated the feasibility of using bacteria to produce immune-activating cyclic dinucleotides in situ, triggering STING (stimulator of interferon genes) pathway activation within tumours and establishing a new class of microbiome-based cancer immunotherapies [151]. At the same time, a preclinical study has shown that engineered *E. coli* can be adapted for tumour-targeted delivery of cytokines such as IL-2, achieving localised immune activation and measurable antitumour effects in vivo [150]. In addition to immunotherapy, recent studies have focused on improving the biomanufacturing capacity and stability of probiotic chassis organisms, which has historically limited translational potential. For instance, next-generation engineering strategies have enabled stable, antibiotic-free, high-level protein expression in *E. coli* Nissle, allowing efficient in situ production of antimicrobial peptides such as microcins and enhancing competitiveness within the gut microbiome [152]. These advances directly address key barriers to clinical deployment, including genetic stability, scalability, and regulatory compatibility.

Taking a broader view, engineered probiotics are now being developed for diverse therapeutic modalities, including oral vaccine delivery and small-molecule biosynthesis. A recent study demonstrates that *E. coli* Nissle can function as an oral delivery platform for tumour-associated antigens, enabling non-invasive vaccination strategies [153]. On the other hand, studies have engineered this strain to biosynthesise complex small-molecule drugs such as romidepsin directly within host-associated environments, highlighting its potential as a living platform for drug production beyond proteins [154]. Concurrently, advances in strain engineering have enabled probiotics to respond to disease-associated physiological cues and release therapeutic molecules dynamically within inflamed tissues, reinforcing their utility as context-responsive therapeutic systems (see review by Duan et al. [155]). All these recent developments broadly highlight a shift from conceptual demonstrations to functionally robust and clinically relevant living therapeutics, in which engineered probiotics act as localised bioreactors capable of sustained, targeted, and disease-responsive drug delivery. By integrating metabolic engineering, improved chassis design, and therapeutic payload diversification, these systems are increasingly positioned to bridge the gap between microbiome science and precision pharmacology.

### 6.3. CRISPR-Based Microbiome Editing

Recent advances in technologies related to CRISPR and associate proteins (CRISPR–Cas3) have enabled precise manipulation of microbial genomes within complex communities. CRISPR-based microbiome editing refers to the use of RNA-guided nucleases to selectively target, modify, or eliminate specific bacterial strains or genes in situ. It enables strain-level precision that is not achievable with conventional broad-spectrum antibiotics. An earlier foundational study demonstrated that CRISPR–Cas nucleases could be programmed to produce sequence-specific antimicrobials capable of selectively killing targeted bacteria while sparing non-target species [156]. This established the conceptual basis for microbiome editing. Over the past five years, major progress has focused on overcoming the key translational barrier of delivering CRISPR systems into microbial populations within the gut environment. Phage-based delivery platforms have emerged as a central strategy for achieving this. For example, phage-delivered CRISPR–Cas3 systems have been used in vivo to selectively target and eliminate *C. difficile*, demonstrating therapeutic potential against clinically relevant pathogens within complex host-associated microbial communities [157]. Similarly, CRISPR–Cas9 delivered via bacteriophages has enabled strain-specific depletion and genomic editing of gut *E. coli*, confirming that precise genetic manipulation of microbiome members is achievable in vivo [158]. Complementing this, broader analyses have highlighted the general applicability of phage-based vectors for delivering CRISPR systems across diverse bacterial taxa, reinforcing their role as a flexible microbiome engineering tool [159].

Engineered probiotic chassis systems have also been developed to enable more controlled and efficient CRISPR delivery directly from commensal bacteria. High-efficiency delivery of CRISPR–Cas9 using engineered probiotic strains has been shown to enable precise microbiome editing with improved stability and targeting efficiency in complex gut environments, expanding beyond phage-only approaches [160]. In addition, conjugative and mobile genetic element-based systems have been explored as alternative dissemination routes for CRISPR machinery, enabling horizontal transfer of editing tools within microbial communities and facilitating selective depletion of antibiotic-resistant populations [161]. Beyond bactericidal applications, CRISPR systems are also being used to modulate microbial function without complete elimination of target organisms. CRISPR interference approaches have been used to stably repress virulence genes and neutralise bacterial pathogenic functions directly within the gut microbiome [162]. This demonstrates that functional reprogramming of microbes is feasible without disrupting community structure. More comprehensively, recent studies have shown that CRISPR-based tools can achieve species- and site-specific genome editing in complex bacterial communities, enabling precise manipulation of microbial genetic content and associated metabolic outputs in vivo [163]. All these advances establish CRISPR-based microbiome editing as a powerful platform for next-generation antimicrobial development and functional microbiome engineering. By enabling precise, programmable, and potentially reversible manipulation of microbial populations, this approach represents a shift from broad-spectrum microbiome modulation toward genotype-directed therapeutic strategies that target disease-relevant genes and pathways within complex microbial ecosystems.

Readers should note that the clinical translation of CRISPR-based microbiome editing is constrained at the moment by regulatory, ecological, and biosafety challenges. Regulatory authorities have not fully adapted to self-replicating live biotherapeutics, which results in uncertainty in approval and long-term monitoring. Ecologically, engineered microbes may behave unpredictably, including loss of function, horizontal gene transfer, and disruption of native microbial networks. Biosafety concerns include persistent colonisation, off-target metabolic effects, and environmental dissemination. Addressing these issues requires long-term surveillance strategies and harmonised regulatory frameworks for engineered living therapeutics.

### 6.4. Microbiome-Responsive Drug Delivery Systems

An alternative strategy in microbiome-based drug discovery exploits microbial activity as an endogenous trigger for site-specific drug release. Microbiome-responsive drug delivery systems are designed to undergo activation in the presence of specific bacterial enzymes or metabolites, enabling spatially restricted pharmacological activity within the gastrointestinal tract. A classic example is the use of azo-bond-containing prodrugs, which are selectively cleaved by bacterial azoreductases in the colon, forming the basis of established colon-targeted therapies [164]. This enzymatic principle remains highly relevant but has now been significantly extended through modern materials chemistry and microbiome-aware drug design. Over the past five years, there has been substantial progress in engineering microbiota-sensitive prodrugs and biomaterials with improved specificity, stability, and responsiveness. The studies have developed azo-linked polymeric prodrugs and nanoparticle systems that remain stable throughout the upper gastrointestinal tract but undergo rapid cleavage in the anaerobic colonic environment where azoreductase activity is enriched. This approach enabled controlled release of anti-inflammatory agents such as 5-aminosalicylic acid with prolonged therapeutic exposure in models of colitis [165,166]. These systems demonstrate that microbial enzymatic activity can be exploited not only for drug activation but also for spatiotemporal tuning of release kinetics to improve local drug concentration while reducing systemic exposure.

Advances in microbiota-sensitive polymer science have also expanded the range of triggers beyond azoreductases to include glycosidases, polysaccharide-degrading enzymes, and microbiota-derived reductive environments. Reviews and experimental studies over the past five years have highlighted natural polysaccharide-based coatings and prodrugs that are selectively degraded by colonic microbiota, enabling highly selective drug release in inflammatory bowel disease and colorectal cancer contexts [167,168]. These systems exploit the elevated enzymatic capacity of the gut microbiome to achieve functionally targeted pharmacokinetics driven by microbial ecology rather than host physiology alone. More recent studies on microbiome-responsive delivery have evolved toward multi-trigger and fail-safe systems, in which drug release is governed by combined pH gradients and microbial enzymatic activity to improve robustness across inter-individual variability in gut microbiota composition. Such dual-responsive systems reduce variability in therapeutic outcome and ensure more reliable colonic delivery across heterogeneous patient populations [169,170,171]. This represents a key translational advance, addressing one of the major limitations of earlier microbiota-triggered systems. All these developments demonstrate that microbiome-responsive drug delivery has evolved from simple azo-bond prodrug activation into a sophisticated class of biologically programmed delivery systems, where microbial enzymatic activity, metabolic environment, and ecological composition are harnessed to achieve precise spatial, temporal, and disease-adaptive drug release. This also positions the gut microbiome not only as a therapeutic target, but also as a functional interface for programmable pharmacology.

### 6.5. Synthetic Microbial Consortia and Ecological Engineering

Beyond single-strain engineering, synthetic ecological approaches aim to design stable microbial consortia with defined functional outputs, where metabolic tasks are distributed across multiple species to enhance robustness, stability, and therapeutic efficiency. An earlier study demonstrated that engineered microbial communities can maintain stable population dynamics while performing coordinated metabolic behaviours, such as sustained metabolite production and division of labour across strains [172]. However, over the past five years, the field has shifted decisively toward therapeutically deployable microbial ecosystems designed to function in vivo within the mammalian gut. Beyond single-strain engineering, synthetic ecological approaches now aim to design stable microbial consortia with defined functional outputs, where metabolic tasks are distributed across multiple species to enhance robustness, stability, and therapeutic efficiency. The study by Venturelli et al. [172] demonstrated that engineered microbial communities can maintain stable population dynamics while performing coordinated metabolic behaviours, such as sustained metabolite production and division of labour across strains. However, the field has now shifted more toward therapeutically deployable microbial ecosystems designed to function in vivo within the mammalian gut.

Recent studies have further shown that rationally designed microbial consortia can restore disrupted metabolic functions associated with disease states. Defined multi-strain communities have been engineered to reconstitute key gut metabolic activities, including SCFA production and bile acid transformation, thereby correcting disease-associated metabolic imbalances in preclinical models of intestinal inflammation and metabolic dysfunction. The foundational study by Goodman et al. [173] demonstrated that microbial community structure itself can restore missing metabolic capabilities through functional redundancy and metabolic complementation. More recent studies have extended these findings to engineered and defined consortia capable of improving host metabolic outputs and epithelial function in vivo, reinforcing the therapeutic potential of distributed metabolic engineering [174,175].

A synthetic consortium has also been conceptualised and experimentally validated as self-organising therapeutic ecosystems, in which community structure and metabolic flux jointly determine functional output. Gut microbial communities are governed by dense metabolic cross-feeding networks that enable emergent stability and collective behaviour [176]. These interaction networks provide a framework for designing consortia in which therapeutic function emerges from ecological organisation rather than single-strain activity. Altogether, these findings establish synthetic microbial consortia as a scalable platform for microbiome-based drug discovery, enabling durable, adaptable, and system-level therapeutic interventions through deliberate ecological engineering of microbial communities. The overall strategic approach of engineered microbiome therapeutics is summarised in Table 4.

## 7. Future Prospects

The field of microbiome-informed drug discovery is rapidly transitioning from descriptive associations toward predictive and engineered system-level pharmacology. Recent studies have demonstrated that microbiome composition and function can be used to predict therapeutic response, particularly in metabolic and immunomodulatory therapies, where baseline microbial features influence outcomes of drugs such as metformin and statins [60,80]. Expanding on this, multi-omics and machine-learning frameworks are now being developed to integrate metagenomic and metabolomic signatures for patient-specific drug response prediction and stratification [177,178]. On the other hand, recent advances in synthetic biology are accelerating the development of programmable microbial systems capable of in situ sensing and therapeutic delivery. First-in-human and advanced preclinical studies using engineered probiotics such as SYNB1891 have demonstrated the feasibility of microbiome-based immunotherapeutic activation via STING pathway signalling within tumours [151]. These developments suggest a shift toward closed-loop living therapeutics that couple environmental sensing with autonomous drug production or immune modulation.

At the level of drug delivery, recent studies in microbiome-responsive biomaterials have extended classical azoreductase-based prodrug activation into multi-trigger systems that integrate microbial enzymatic activity with pH and redox sensitivity, improving robustness across heterogeneous gut environments [165,169]. Such approaches are ever more being designed to reduce inter-individual variability in drug release profiles, a major limitation of earlier microbiota-triggered systems. Synthetic microbial consortia are also moving toward clinically relevant applications. Defined multi-strain therapeutics such as VE303 have demonstrated that rationally designed communities can achieve predictable colonisation and durable protection against recurrent *C. difficile* infection in clinical trials [22,29]. These findings support a broader shift from donor-derived variability toward standardised, ecologically engineered microbial medicines.

Finally, CRISPR-based microbiome editing continues to mature as a precision tool for selective microbial modulation. Recent in vivo studies using phage-delivered CRISPR systems have demonstrated strain-specific targeting and gene-level editing within complex gut communities, highlighting the potential for reversible and programmable microbiome control [146,147,152]. All these advances indicate that microbiome-aware drug discovery is evolving toward a predictive and programmable discipline, in which microbial ecology is not only measured but actively engineered to optimise therapeutic outcomes.

The unified pharmacology framework presented herein linking clinical drugs, natural products, and engineered microbial therapeutics represents a promising future direction for precision medicine and systems pharmacology. Key stakeholders who would benefit include pharmaceutical and biotechnology industries through improved prediction of drug efficacy, safety, and microbiome-mediated variability; regulatory agencies through enhanced frameworks for evaluating microbiome-dependent drug responses and safety assessment; healthcare systems and clinicians via improved patient stratification and microbiome-informed therapeutic decision-making; and developers of microbiome-based therapies such as FMT and live biotherapeutics through rational, mechanism-guided design of living medicines. Implementation of this framework will require standardisation of microbiome and multi-omics data collection, incorporation of microbiome variables into pharmacokinetic/pharmacodynamic modelling, development of predictive computational tools, establishment of harmonised regulatory guidelines for microbiome-informed therapeutics, and large-scale longitudinal clinical studies to validate causality and clinical utility.

## 8. Conclusions

The gut microbiome fundamentally reshapes contemporary drug discovery by acting as both a biochemical transformer of xenobiotics and an active therapeutic target. The four-layer framework presented here captures this continuum, from ecosystem-dependent interventions such as FMT, through microbiome-modulated approved drugs and microbiota-dependent natural products, to fully engineered microbial therapeutics. Across these layers, a unifying principle emerges: drug efficacy, toxicity, and bioavailability are frequently emerging properties of host–microbiome co-metabolism rather than host biology alone. This challenges traditional pharmacological paradigms centred on single-organism or single-target models and instead positions the microbiome as a dynamic, system-level determinant of therapeutic outcome.

The progression toward engineered microbiome therapeutics further demonstrates that microbial communities can be rationally designed to sense, respond, and act within the host environment. This represents a conceptual shift from pharmacology in the host to pharmacology within a coupled host–microbiome system. Overall, integrating microbiome science into drug discovery provides a unified framework for understanding variability in drug response and opens new avenues for precision therapeutics, living medicines, and ecosystem-level interventions.

## Figures and Tables

**Table 1:** Exemplary therapies whose efficacy depends on an intact, functionally competent gut microbiome *.

Sub-Category	Therapeutic Modality	Mechanistic Basis	Microbiome Dependency Level	Key Clinical Applications	Key References
Ecosystem restoration therapy—serving as gold standard	FMT	Restoration of colonisation resistance, bile acid metabolism, SCFA production, and immune homeostasis via full microbial community transfer	Complete dependency on intact, diverse ecosystem	Recurrent *Clostridioides difficile* infection (high cure rates), emerging: IBD, metabolic disease	[13,14,15]
Standardised microbiome replacement products	Live biotherapeutic products derived from FMT (e.g., RBX2660, SER-109/Vowst)	Defined or semi-defined microbial communities restore ecological balance and bile acid metabolism; improved reproducibility versus donor FMT	High dependency (ecosystem reconstruction, reduced complexity)	Recurrent *C. difficile* infection prevention	[20,22,23]
Defined microbial consortia-next-generation FMT derivatives	Rational or purified multi-strain consortia (e.g., VE303)	Engineered or selected strains restore key ecological functions (colonisation resistance, bile acid transformation) via competitive exclusion	High but controlled dependency—minimal ecosystem reconstruction	Recurrent *C. difficile* infection	[29,30,34]
Donor-dependent microbiome modulation in complex disease (experimental FMT expansion)	FMT in metabolic, inflammatory, and neuroimmune disorders	Microbiome transfer alters host immunity, metabolism, and neuroinflammation via undefined community effects	High but unstable dependency (variable engraftment)	T2D, IBD, cancer immunotherapy response, neurodegenerative diseases	[43,44,46,47,50]
Postbiotic/cell-derived functional therapies (transition zone)	Microbial metabolites and inactivated bacteria (e.g., SCFAs, pasteurised *Akkermansia muciniphila*)	Defined microbial components recapitulate functional outputs without live ecosystem transfer	Functional dependency (loss of ecosystem requirement, retention of microbial signalling)	Metabolic disease, inflammation, barrier dysfunction	[40,41]

* Modalities include faecal microbiota transplantation (FMT), defined microbial consortia, live biotherapeutic products, and postbiotic approaches. Therapeutic effects involve restoration of colonisation resistance, bile acid metabolism, short-chain fatty acid (SCFA) production, and immune modulation, with representative evidence from recurrent *Clostridioides difficile* infection and emerging metabolic and inflammatory disease applications. IBD, inflammatory bowel disease; T2D, type-2 diabetes; SCFAs, short-chain fatty acids.

**Table 2:** Microbiome-modulated approved drugs: mechanistic classes and clinical consequences *.

Therapeutic Class	Drug Example(s)	Microbiome Mechanism	Type of Modulation	Key Microbial Process/Pathway	Clinical Consequence	Key References
Antidiabetic agents	Metformin	Microbiome-driven reshaping of gut ecology and metabolite production	Indirect efficacy enhancement	↑ *Akkermansia muciniphila*, SCFA production, bile acid signalling (FXR/TGR5), GLP-1 modulation	Improved insulin sensitivity and glucose homeostasis	[55,58,59,60]
Chemotherapeutics—toxicity modulation	Irinotecan	Microbial reactivation of drug metabolites	Toxicity amplification	β-glucuronidase-mediated SN-38 reactivation in gut lumen	Severe diarrhoea, dose-limiting toxicity	[61,62,63,65]
Neurological agents	Levodopa (L-DOPA)	Microbial enzymatic degradation prior to absorption	Reduced efficacy/bioavailability loss	*Enterococcus faecalis* decarboxylation; *Eggerthella lenta* metabolism	Reduced CNS drug availability; interpatient variability in PD therapy	[67,68]
Cardiovascular drugs	Digoxin	Microbial enzymatic inactivation	Drug inactivation	*Eggerthella lenta* reductase pathway; diet-dependent regulation	Reduced circulating active drug levels	[69]
Anti-inflammatory/IBD drugs	Sulfasalazine	Microbial prodrug activation	Required for efficacy	Bacterial azoreductase cleavage → 5-aminosalicylic acid activation	Therapeutic activation in colon	[70,71]
Lipid-lowering agents	Statins (simvastatin, rosuvastatin)	Microbiome-dependent modulation of metabolism and bile acids	Variable efficacy	Microbiome-driven bile acid metabolism and host–microbe co-metabolism	Inter-individual variability in LDL reduction	[72,73]
Chemotherapeutics—immune modulation	Cyclophosphamide	Microbiome-mediated immune activation	Enhanced anticancer efficacy	Gut bacteria promote Th1/Th17 polarisation	Improved antitumour immune response	[74]
Antimetabolite chemotherapies	5-Fluorouracil	Microbiome-driven modulation of toxicity	Toxicity modulation	Microbial–mucosal interactions influencing inflammation and metabolism	Altered GI toxicity and treatment response	[76,77,78]
Antibiotics—microbiome-disrupting drugs	Clindamycin, β-lactams	Broad microbiome depletion and dysbiosis	Indirect disease susceptibility	Loss of colonisation resistance, altered community structure	Secondary infections (e.g., *C. difficile*)	[14]
Non-GI systemic drugs—emerging class	Acetaminophen, sulfonamides	Microbiome-mediated co-metabolism affecting pharmacokinetics	Variable biodisposition	Microbial metabolite interaction with hepatic pathways	Altered systemic exposure and clearance	[79]

* Drugs are grouped by interaction type, including enhanced efficacy, prodrug activation, drug inactivation, toxicity modulation, and response variability. Key microbial mechanisms and representative clinical outcomes highlight the microbiome’s role in shaping drug disposition and therapeutic response. GI, gastrointestinal; FXR, farnesoid X receptor; GLP-1, glucagon-like peptide-1; IBD, inflammatory bowel disease TGR5; Takeda G-protein-coupled receptor 5.

**Table 3:** Major dietary and plant-derived compounds whose biological activity depends on gut microbiota-mediated biotransformation *.

Natural Product Class	Key Microbiota-Driven Transformation	Bioactive Metabolite(s)	Functional/Physiological Outcome	Key References
Polyphenols (flavonoids, tannins, curcumin, resveratrol, epigallocatechin-3-gallate)	Deglycosylation, ring fission, reduction, dehydroxylation	Phenolic acids, urolithins, dihydroresveratrol, valerolactones	Improved bioavailability; antioxidant, anti-inflammatory, metabolic effects	[85,87,88,89,94,95]
Ginsenosides	Stepwise microbial deglycosylation (β-glucosidases)	Ginsenoside compound K	Enhanced anti-inflammatory, anticancer activity; increased absorption	[100,101,102,103]
Alkaloids—berberine and others	Microbial reduction (nitroreductase activity)	Dihydroberberine	Increased lipophilicity and intestinal absorption; metabolic effects	[107,108,110]
Dietary fibre (resistant starch, inulin)	Anaerobic fermentation and cross-feeding	SCFAs—acetate, propionate, butyrate	Energy regulation, anti-inflammatory effects, epigenetic modulation	[113,114,115,116]
Isoflavones (e.g., daidzein)	Reductive metabolism via intermediate dihydrodaidzein pathways	Equol	Enhanced estrogenic and cardiometabolic effects; inter-individual variability (equol producer phenotype)	[123,125,126,127]
Lignans (secoisolariciresinol diglucoside)	Sequential microbial demethylation, dehydroxylation, reduction	Enterodiol, enterolactone	Weak estrogenic activity; cardiometabolic and potential cancer-protective effects	[130,131]
Glucosinolates (cruciferous vegetables)	Microbial hydrolysis of thioglucosidic bonds	Isothiocyanates (e.g., sulforaphane)	Detoxification enzyme induction; chemopreventive effects	[135,136,137,138]

* Microbial processes such as deglycosylation, reduction, hydrolysis, and ring fission generate absorbable or bioactive metabolites with altered pharmacological effects. Examples include polyphenols, ginsenosides, alkaloids, dietary fibres, isoflavones, lignans, and glucosinolates, highlighting the gut microbiome as a key determinant of natural product activity.

**Table 4:** Engineered microbiome therapeutic strategies, including programmable microbes, engineered probiotics, CRISPR-based editing, microbiome-responsive drug delivery, and synthetic consortia *.

Subdomain	Core Engineering Strategy	Representative Systems and Examples	Key Therapeutic or Functional Output	Key References
Programmable microbial systems and synthetic gene circuits	Genetic circuits converting physiological inputs into programmable outputs; biosensing, memory recording, and population control	Tumour-targeting engineered bacteria; synchronised lysis circuits; inflammatory biosensors	In vivo biosensing, disease recording, controlled therapeutic release, drug discovery readouts	[142,143,144,145]
Engineered probiotics as living therapeutics	Metabolic engineering of commensal strains for in situ drug production or substrate depletion	*E. coli* Nissle (phenylalanine depletion); IL-10 secreting *Lactococcus lactis*; IL-2–producing *E. coli*; SYNB1891; VE303; engineered biosynthetic probiotics	Metabolic correction, immune modulation, local cytokine delivery, tumour immunotherapy, antimicrobial peptide production	[148,149,150,151]
CRISPR-based microbiome editing	RNA-guided nucleases delivered via phages, conjugation, or engineered probiotics for strain-level editing	Phage CRISPR–Cas3 targeting *C. difficile*; Cas9 phage editing of gut *E. coli*; CRISPRi repression of virulence genes	Selective pathogen depletion, gene knockouts, virulence silencing, microbiome reprogramming	[156,157,158,160,162,163]
Microbiome-responsive drug delivery systems	Enzyme-, pH-, and metabolite-triggered polymer/prodrug systems for site-specific release	Azo-bond prodrugs; azoreductase-responsive polymers; polysaccharide-degradable coatings; dual-trigger systems	Colon-targeted drug release, reduced systemic toxicity, controlled pharmacokinetics	[164,165,166,167,169,170]
Synthetic microbial consortia and ecological engineering	Multi-strain communities with distributed metabolic tasks and engineered ecological stability	Defined SCFA-producing consortia; bile acid-modulating communities; VE303-like designs; stable colonising consortia	Restoration of metabolic function, SCFA production, bile acid metabolism, epithelial barrier repair	[172,173,174,176]

* These approaches use microbes as engineered platforms for sensing, drug production, delivery, and ecological reprogramming. The table summarises key mechanisms, engineering principles, and representative evidence, highlighting a shift toward programmable, context-responsive microbiome therapeutics.

## Data Availability

No new data were created or analysed in this study.
